# Preliminary exploration of the co-regulation of Alzheimer’s disease pathogenic genes by microRNAs and transcription factors

**DOI:** 10.3389/fnagi.2022.1069606

**Published:** 2022-12-06

**Authors:** Qi Zhang, Ping Yang, Xinping Pang, Wenbo Guo, Yue Sun, Yanyu Wei, Chaoyang Pang

**Affiliations:** ^1^School of Computer Science, Sichuan Normal University, Chengdu, China; ^2^West China School of Basic Medical Sciences and Forensic Medicine, Sichuan University, Chengdu, China; ^3^National Key Laboratory of Science and Technology on Vacuum Electronics, School of Electronic Science and Engineering, University of Electronic Science and Technology of China, Chengdu, China

**Keywords:** Alzheimer’s disease (AD), differentially expressed genes (DEGs), COX regression, transcription factors, miRNAs

## Abstract

**Background:**

Alzheimer’s disease (AD) is the most common form of age-related neurodegenerative disease. Unfortunately, due to the complexity of pathological types and clinical heterogeneity of AD, there is a lack of satisfactory treatment for AD. Previous studies have shown that microRNAs and transcription factors can modulate genes associated with AD, but the underlying pathophysiology remains unclear.

**Methods:**

The datasets GSE1297 and GSE5281 were downloaded from the gene expression omnibus (GEO) database and analyzed to obtain the differentially expressed genes (DEGs) through the “R” language “limma” package. The GSE1297 dataset was analyzed by weighted correlation network analysis (WGCNA), and the key gene modules were selected. Next, gene ontology (GO) and Kyoto encyclopedia of genes and genomes (KEGG) pathway enrichment analysis for the key gene modules were performed. Then, the protein-protein interaction (PPI) network was constructed and the hub genes were identified using the STRING database and Cytoscape software. Finally, for the GSE150693 dataset, the “R” package “survivation” was used to integrate the data of survival time, AD transformation status and 35 characteristics, and the key microRNAs (miRNAs) were selected by Cox method. We also performed regression analysis using least absolute shrinkage and selection operator (Lasso)-Cox to construct and validate prognostic features associated with the four key genes using different databases. We also tried to find drugs targeting key genes through DrugBank database.

**Results:**

GO and KEGG enrichment analysis showed that DEGs were mainly enriched in pathways regulating chemical synaptic transmission, glutamatergic synapses and Huntington’s disease. In addition, 10 hub genes were selected from the PPI network by using the algorithm Between Centrality. Then, four core genes (TBP, CDK7, GRM5, and GRIA1) were selected by correlation with clinical information, and the established model had very good prognosis in different databases. Finally, hsa-miR-425-5p and hsa-miR-186-5p were determined by COX regression, AD transformation status and aberrant miRNAs.

**Conclusion:**

In conclusion, we tried to construct a network in which miRNAs and transcription factors jointly regulate pathogenic genes, and described the process that abnormal miRNAs and abnormal transcription factors TBP and CDK7 jointly regulate the transcription of AD central genes GRM5 and GRIA1. The insights gained from this study offer the potential AD biomarkers, which may be of assistance to the diagnose and therapy of AD.

## Introduction

Alzheimer’s disease (AD) is the most common and complex neurological disease in the world. Neurological conditions such as AD are the leading cause of 70% of dementia worldwide (Mayeux and Stern, 2012). Global Alzheimer’s cases are increasing year by year and are expected to reach 78 million by 2030. While the pathogenesis of the disease is not yet known, many believe the accumulation of over-phosphorylated tau and Aβ plaques in neurofibrillary tangles are the main causes of AD (Andreasen et al., 1999; Shao et al., 2011; Arvanitakis and Bennett, 2019). When extracellular amyloid plaques build up in specific areas of the brain, they can lead to amyloid vascular disease or neurodegenerative diseases (Thanvi and Robinson, 2006; Jäkel et al., 2022). The neurofibrillary tangles (NFTs) are huge paired intracellular helical strands of hyperphosphorylated tau proteins, which induce neuronal and synaptic loss (Moloney et al., 2021). The predominant regions of the pathological process underlying AD in human brain are the association areas of the cerebral cortex and the hippocampus (Wang et al., 2010). Despite these extensive findings on both, there are few effective drugs to improve and treat AD. Worse, many patients have to wait a long time to be diagnosed with the disease. During this period, the Aβ burden is significant and memory loss has already occurred (Arvanitakis et al., 2019; Long and Holtzman, 2019). Typical AD has gone through a gradual and hidden development process, and there is no specific detection method, which cannot be accurately diagnosed, difficult to cure, difficult to control, and poor prognosis. Therefore, it is urgent to explore new potential biomarkers for early diagnosis and effective treatment of AD.

Alzheimer’s disease is the most common neurodegenerative disorder with limited therapeutics, and AD is characterized by the formation of plaques made by protein aggregates. Mounting studies have suggested that targeting transcription factors holds promise for treating neurodegenerative disorders including AD (Ping Yang et al., 2022). CMV promotor-driven transcription factor EB (TFEB), for example, is injected with adeno-associated viral particles in targeted mice, which are mainly localized in neuronal nuclei and upregulated lysosomes. This resulted in decreased steady state levels of Aβ in APP proteins and cerebral stromal liquid (Xiao et al., 2015; Song et al., 2020). A growing body of evidence also suggests that the accumulation of misfolding proteins in AD is caused by damage to macromolecular autophagy and autophagy lysosomal pathways, making TFEB, which regulates autophagic lysosomal pathway, a promising target for AD treatment (Zheng et al., 2021). We also found decreased expression of the transcription factor Nrf2 and its NQO1, HO-1, and GCLC driver genes, and changes in related Nrf2 pathways in AD brains. Nrf2 activation may provide cellular protection and prevent an increasing number of diseases including neurodegenerative diseases. These avenues of evidence point to the activation of the Nrf2 transcription factor as a potential new therapeutic scheme for AD (Osama et al., 2020). Additional research shows that ATF6 transcription factor activation can attenuate amyloidosis through BACE1 downregulation, and concomitantly ATF6 overexpression significantly reduces Aβ1-42. The results of this study suggest ATF6 may become a potential focus for targeted therapy of AD. Interestingly, TBP and CDk7 also belong to the basal transcription factor family like ATF6 among the 10 key genes selected in our study (Geng et al., 2016; Du et al., 2020).

Recently, progress has begun to clarify the physiological and pathological roles of non-coding RNAs (ncRNAs) in a variety of diseases, including cancer. ncRNAs do not have protein coding functions, but are important regulators of many cellular processes (Esteller, 2011). Of these, miRNAs are the most studied and have become key players in the pathogenesis of AD involved in regulating key growth regulatory pathways (Swarbrick et al., 2019; Toden et al., 2021). miRNAs are small endogenous non-coding RNA molecules that can inhibit or silence the expression of post-transcription genes. Many miRNAs play an important role in post-transcriptional regulation and are highly conserved (Sand et al., 2009). Previous research has found that numerous miRNAs, including miRNA-126a-3p, MicroRNA-455-3p, miR-501-3p, and miRNA-101a-3p, have a role in the pathogenesis of AD, showing that miRNAs are strongly linked to the development of AD (Hara et al., 2017; Kumar et al., 2017; Kumar and Reddy, 2019; Lin et al., 2022; Xue et al., 2022). However, the specific role and potential mechanisms of miRNA in AD pathogenesis are unclear.

We here established a miRNA-mRNA, TF-mRNAs regulatory network by integrating relevant TFs (transcription factors), miRNAs and mRNAs to try to gain further insight into the potential functions of ncRNAs as well as transcription factors in AD. This study may provide a better understanding of the underlying pathogenesis of AD and potentially new biomarkers for diagnosis and treatment of AD.

## Materials and methods

### Gene expression profile data collection

The gene expression datasets used in this investigation were collected from the gene expression omnibus (GEO) database.^1^ This database generated a total of 340 datasets on human AD. After a careful review, four gene expression profiles (GSE5281, GSE1297, GSE122063, and GSE150693) were selected. Among them, GSE5281 was based on the GPL570 platform ([HG-U133_Plus_2] Affymetrix Human Genome U133 Plus 2.0 Array), GSE1297 was built on GPL96 Affymetrix Human Genome U133A Array. All data were freely accessible online. The GSE5281 expression profile consists of 161 samples and approximately 55,000 transcripts from 74 disease-free patients and 84 patients with AD. There are 31 samples in the GSE1297 dataset, including 9 normal and 22 AD subjects with different severities. The correlation between each gene expression and clinical information of the 31 subjects was verified by the Neurofibrillary Entanglement Score (NFT) and the Minimental Examination (MMSE) (Blalock et al., 2004; Liang et al., 2007). GSE122063 is a gene expression profiling in frontal and temporal cortices from 36 patients with vascular dementia, 56 diseases (AD), and 44 non-demented controls (Control) obtained from the University of Michigan. Brain Bank Mild cognitive impairment (MCI) is a precursor to the development of AD. The GSE150693 data were downloaded from the GEO database from the GPL21263 platform, among them 197 miRNA samples from MCI sera included 83 patients who were converted from MCIs to AD, and 114 patients who did not convert from MCI to AD (Shigemizu et al., 2020).

### Application of weighted correlation network analysis algorithm

First, the GSE1297 sample data obtained from the GEO database was processed by using the log scale robust multi-array analysis (RMA). Then the missing values in the samples were filled by using the K-neighborhood algorithm to improve the accuracy and usability of the data. Next, expression relationships among genes were measured by correlation coefficients to construct weighted co-expression networks in the absence of outlier samples through cluster analysis of gene samples. Based on this, gene modules were identified by using a clustering algorithm as well as a dynamic shear tree algorithm. The calculated correlation coefficients between gene modules and clinical performance traits were used as the association criteria. Using the Pearson correlation test can analyze the relationship between module eigengenes and clinical features, and select the modules for focused mining. Among them, clinical information includes: Age, age at death; NFT, neurofibrillary tangle count; Braak, Braak stage; MMSE, adjusted Minimental Status Exam; PMI, postmortem interval. Finally, the key modules were further mined to find the key core genes.

### Functional classification and pathway enrichment of differentially expressed genes

The above differentially expressed genes (DEGs) screened from the weighted correlation network analysis (WGCNA) were submitted to gene ontology (GO) function enrichment analysis, which was consisted of cellular component (CC), biological process (BP), and molecular function (MF), and was analyzed and visualized by using the R language “cluster profile,” “GGploT2,” and “enrichPlot” packages. Kyoto encyclopedia of genes and genomes (KEGG) pathway enrichment analysis of DEGs in this study was performed by the Database for Annotation, Visualization, and Integrated Discovery (DAVID) tools.^2^ KEGG pathway visualization was analyzed by Sangerbox online pathway analysis tool (Shen et al., 2022). Enriched GO terms with adjusted value *P* < 0.01 and gene number> 12 were selected, and *P* > 0.05 of KEGG pathways and gene numbers> 5 were considered statistically significant.

### Analyzing differential expression

DEGs are processed using the “R” language “limma” package and calculate adjusted P values and | logFC|. For GSE5281 and GSE97760 gene expression profiles, Log2 Fold Change (FC) | > 1.0 and adjusted *P*-values < 0.05 were selected as cut-off criteria.

### Protein-protein interaction network and module analysis

WGCNA was used to directly set thresholds for gene saliency (GS) and module feature (MM). Based on | MM| > 0.8, | GS| > 0.1 threshold value for the standard to filter core genes, 269 key genes were selected from 789 genes in the “midnightblue” module. To explore the relationship between these 269 hub genes and MAPT (AD is defined by the presence of extracellular and intracellular neurofibrillary tangles composed of hyperphosphorylated tau proteins) and AD disease (Corbo and Alonso Adel, 2011; Bakota and Brandt, 2016; Chong et al., 2018), the Search Tool was used for the Retrieval of Interacting Genes (STRING version 11.5) for known or predicted, direct (physical) and indirect (functional) PPIs (Szklarczyk et al., 2015). The interaction confidence score of >0.4 was used as the cutoff value for statistical significance. Subsequently, the PPI network was visualized by Cytoscape3.9 software.^3^ To better search for proteins related to Tau protein (MAPT), the algorithm between Centrality was adopted in this study to find the important nodes [the calculation formula is (1) and (2)].

We used CytoHubba, a plugin in Cytoscape, to compute nodes in the protein network that efficiently convey data. In our study, the top 10 genes were identified as central genes.


(1) $$C_{B}(N_{i})=\sum_{k,j=1}^{g}sd\left(k,i,j\right),\left(k\neq j\right)$$

sd(k, i, j) indicates that the shortest path from k to j passes through i, which means, i is on the shortest path from k to j.


(2) $$C_{B}^{\prime }\left(N_{i}\right)=\frac{C_{B}\,\left(N_{i}\right)}{\sum_{k,j=1}^{g}\left(k,j\right)},\left(k\neq j\right)$$

The formula can be standardized to obtain (2), where the denominator represents the number of paths between two points in the figure, that is, the number of all paths.

### Gene set enrichment analysis

For Gene Set Enrichment Analysis (GSEA), we obtained data from GSEA (DOI: 10.1073/pans.0506580102)^4^ website for the GSEA software (version 3.0), the samples were divided into high expression group (≤50%) and low expression group (<50%) according to the expression level of GRM5 and GRIA1. Obtained from the Molecular Signatures Database (DOI: 10.1093/bioinformatics/btr260),^5^ a subset of “c3.mir.v7.4.symbols.gmt” and “c3.tft.v7.4.symbols.gmt” was downloaded to evaluate related pathways and molecular mechanisms, based on gene expression profile and phenotype grouping. The minimum gene set was 5. The maximum gene set was 5,000, 1,000 resampling, *P*-value < 0.05 and FDR < 0.25 were considered statistically significant, to verify the binding relationship of miRNAs and transcription factors with the target genes, and to indicate that they are on the relevant biological pathway.

### MicroRNAs associated with hub

#### Genes

The ENCORI (The Encyclopedia of RNA Interacts) database is used to construct Hub genes and target miRNA interactions with them. We then used the “R” software package “Survival” to integrate data on survival, AD transformation status, and 35 features, and evaluated the prognostic significance of these features in 197 samples by Cox and selection of key microRNAs (miRNAs). Finally, these central genes and miRNA networks were mapped using Cytoscape software.

#### The least absolute shrinkage and selection operator logistic regression algorithm model was constructed

Here, we used the R package “glmnet” to integrate AD status and gene expression data from GSE5281 database for regression analysis using least absolute shrinkage and selection operator (Lasso)-Cox. Tenfold cross validation was performed to obtain the optimal model. We set the Lambda value to 0.00208765767322578 and finally obtained four genes. The model formula is: RiskScore = −0.00111259701091014 × TBP-0.00540566228324965 × CDK7 + 0.0013635456849925 × GRM5 − 0.000512469864405875 × GRIA1 and then, the database GSE122063 was used to verify the model to ensure the rationality of the model.

#### The key genes obtained from network analysis were verified with FpClass tool

The FpClass for predicting high confidence PPIs on a proteome-wide scale, including proteins with few (low-degree proteins) or no known binding partners (orphans). To identify co-expressed genes with the queried genes, the tool considers two specific scores: the gene expression score and the topological network score. The gene expression score is based on the Pearson correlation of the gene expression pattern. The topology score of the network shows whether genes exist in the training data and the strength of their interaction (Kotlyar et al., 2015; Sriroopreddy et al., 2019).

#### Drug selection of hub gene

Drugs targeting key genes were retrieved from the DrugBank database, which is a web-enabled database.^6^ The database collects more than 7,800 drugs, including interactions and their targets, drug binding data, drug-drug and drug-food interaction data. It also includes hundreds of drug impact information on metabolite levels, protein expression levels, hundreds of drug clinical trials and drug reuse trials (Wishart et al., 2018).

## Results

### Gene co-expression modules

**FIGURE 1:**
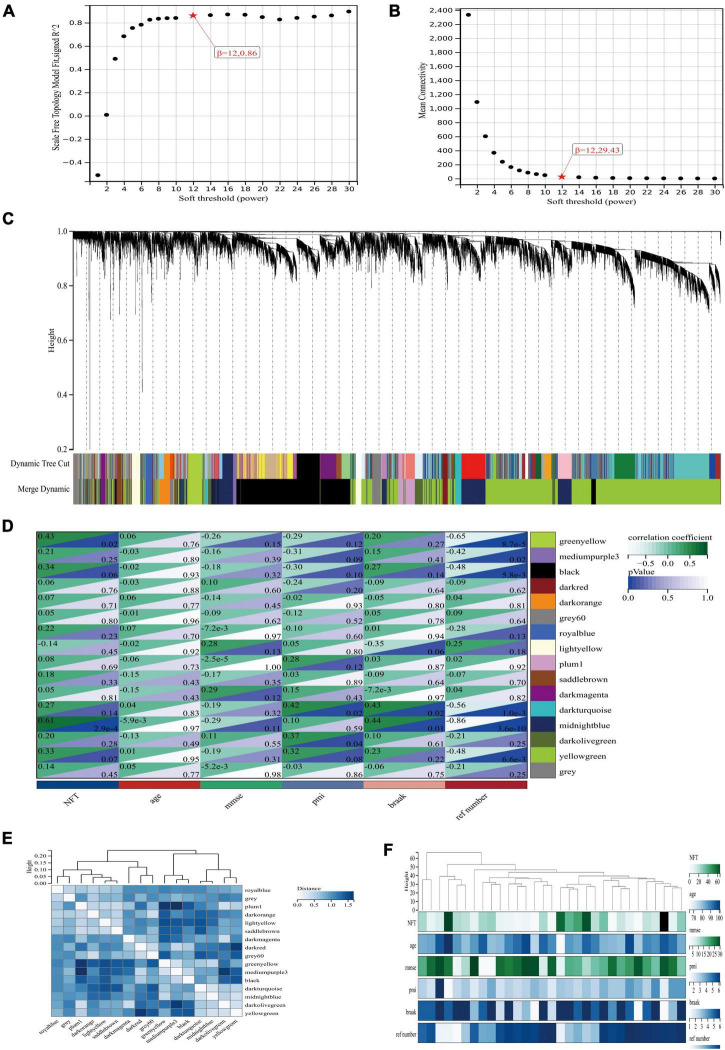
Co-expression network of differentially expressed genes in AD. **(A,B)** Soft threshold power selection. **(C)** Clustering tree dendrogram of co-expression modules. Different colors represent distinct co-expression modules. **(D)** Correlation analysis between midnightblue module and clinical condition, each column represents one module, individual rows represent clinical status. **(E)** Correlation between modules. **(F)** Specific clinical information of the sample.

**FIGURE 2:**
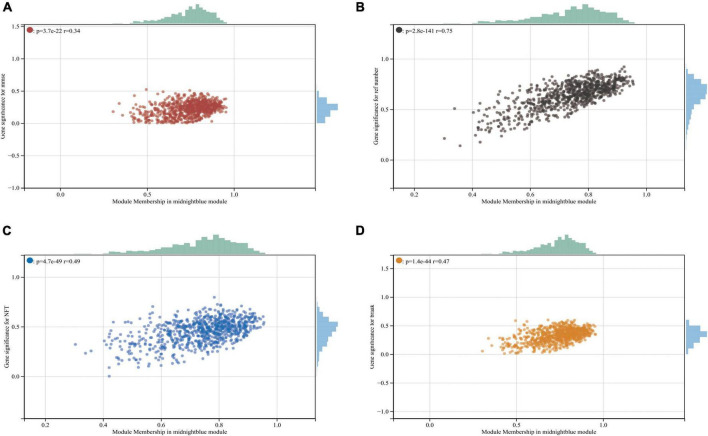
Midnightblue module and clinical relevance. **(A)** Scatter plot between module membership in midnightblue module and the clinical information MMSE. **(B)** Scatter plot between module membership in midnightblue module and clinical information ref number. **(C,D)** Scatter plot between module membership in midnightblue module and clinical information NFT and break, respectively.

To explore the co-expression patterns of mRNA in AD, we performed WGCNA analysis on the GSE1297 dataset. The dataset contained 22 columns of AD samples and 9 columns of normal human samples, with a total of 22,283 genes. The raw data can be downloaded from the GEO database of NCBI.^7^ The read-in data was first preprocessed by the rma function in the Affy package in R language, which contains background correction, normalization, calculation of expression to normalize the data. Then, for the missing values in the data, we used the K-neighborhood algorithm (KNN) to complement the missing values data. Through this algorithm, genes with high similarity were identified to supplement the null values. The absolute median difference (MAD) is the median of the difference between each data in a set of data and the median of that set of data. To filter out a large number of genes with relatively constant expression in different gene samples, the top 7,200 genes of MAD values were selected for WGCNA analysis in this paper. To explore the co-expression patterns of mRNA in AD, we performed WGCNA analysis on the GSE1297 dataset (Figures 1D,F). To ensure scale-free networks, we chose soft thresholds of *b* = 12 (Figures 1A,B), used WGCNA packages as soft threshold power to generate hierarchical clustering trees (Figure 1C), and then we built co-expression networks of associations between clinical features and these modules. As shown in Figure 2 the “Midnight” module of GSE1297 is significantly related to the clinical features of AD.

### Kyoto encyclopedia of genes and genomes pathway and gene ontology analysis of differentially expressed genes

**FIGURE 3:**
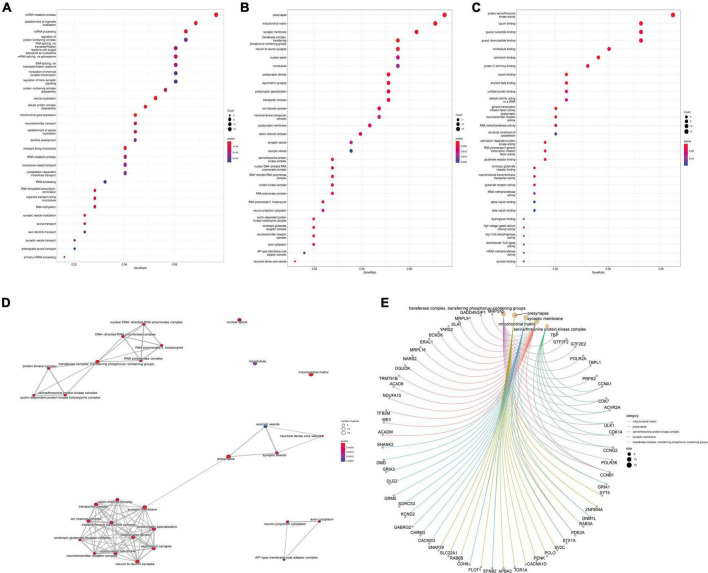
Differentially expressed gene’s (DEG’s) GO analysis. **(A)** GO bioprocess enrichment. **(B)** Cell composition enrichment results. **(C)** Results of molecular function enrichment. The color of each bubble represents the fitted *p*-value: the redder the color, the higher the concentration. **(D)** The relationship between the classes. **(E)** The genes contained in the more enriched category. GO, Gene Ontology.

**FIGURE 4:**
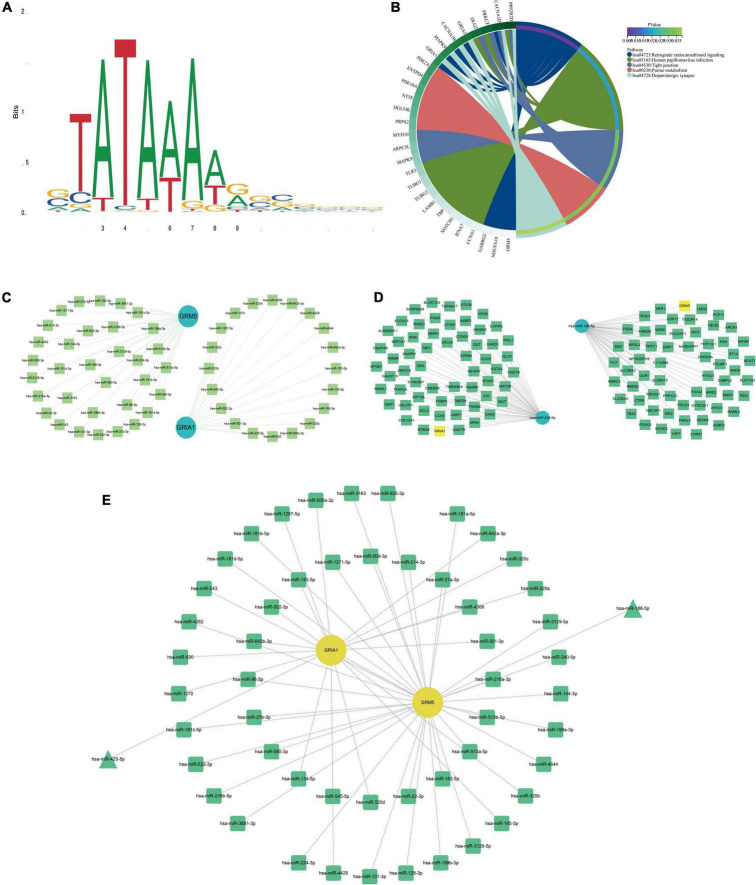
Kyoto encyclopedia of genes and genomes (KEGG) pathway map, TBP binding site map, and key mRNA miRNAs prediction. **(A)** TBP transcription factor binding site. **(B)** KEGG pathway diagrams of differentially expressed genes (DEGs). **(C)** The miRNAs to GRM5 and GRIA1 bind are predicted. In the network, thin lines represent sequence matches, green balls represent miRNAs and blue balls represent mRNA. **(D)** The mRNA binding to hsa-miR-425-5p and hsa-miR-186-5p was predicted. In the network, thin lines represent sequence matching, green spheres represent mRNA, and blue spheres represent miRNA. **(E)** The network diagram of Hub gene and miRNA. The yellow ball represents the gene, the green represents the miRNA, and the green triangle is the miRNA we finally screened.

GO analysis of DEGs (including molecular function, biological processes and cell composition analysis) and KEGG pathway analysis, respectively, can provide valuable evidence of processes and pathways in which gene sets may be involved. This type of information is crucial for hypothesis development and for the design of future research. Functional enrichment analyses of KEGG pathway enrichment and DEGs GO function enrichment analysis for DEGs were performed using the DAVID And R package “cluster profile” (Tables 1, 2). The enriched GO terms were divided into CC, BP, and MF ontologies. The results of GO analysis indicated that DEGs were mainly enriched in BPs, including modulation of chemical synaptic transmission, regulation of trans-synaptic signaling, regulation of supramolecular fiber organization, regulation of membrane potential, vesicle localization, the establishment of organelle localization, protein-containing complex disassembly and regulation of protein-containing complex assembly. MF analysis showed that the DEGs were protein serine/threonine kinase activity, tubulin binding, guanyl nucleotide binding, and geranyl ribonucleotide binding. For the cell components, the DEGs were synaptic membrane, presynapse, mitochondrial matrix, transporter complex, neuron-to-neuron synapse, microtubule, and microtubule. In addition, the results of KEGG pathway analysis showed that DEGs were mainly enriched in pathways in retrograde endocannabinoid signaling, human papillomavirus infection, tight junction, purine metabolism, and dopaminergic synapse (Figures 3, 4B and Supplementary Data 1).

**TABLE 1:** Significantly enriched gene ontology (GO) terms of differentially expressed genes (DEGs).

Category	Term	Description	*P*-value	Count
BP term	GO:0051648	Vesicle localization	0.00000495	13
BP term	GO:0051656	Establishment of organelle localization	0.0000306	17
BP term	GO:0032984	Protein-containing complex disassembly	0.0000637	14
BP term	GO:0043254	Regulation of protein-containing complex assembly	0.00015812	16
BP term	GO:0050804	Modulation of chemical synaptic transmission	0.000179862	15
BP term	GO:0099177	Regulation of trans-synaptic signaling	0.000184724	15
BP term	GO:1902903	Regulation of supramolecular fiber organization	0.000237205	14
BP term	GO:0042391	Regulation of membrane potential	0.00034946	15
CC term	GO:0098793	Presynapse	0.0000018	19
CC term	GO:0097060	Synaptic membrane	0.0000146	16
CC term	GO:0005759	Mitochondrial matrix	0.0000279	18
CC term	GO:1990351	Transporter complex	0.000311367	13
CC term	GO:0098984	Neuron to neuron synapse	0.000348414	13
CC term	GO:0005874	Microtubule	0.000822817	14
CC term	GO:0043025	Neuronal cell body	0.002387617	14
MF term	GO:0004674	Protein serine/threonine kinase activity	0.000143327	16
MF term	GO:0015631	Tubulin binding	0.000968776	13
MF term	GO:0019001	Guanyl nucleotide binding	0.001949535	13
MF term	GO:0032561	Geranyl ribonucleotide binding	0.001949535	13

**TABLE 2:** Significant enrichment of the Kyoto encyclopedia of genes and genomes (KEGG) pathway.

Category	Term	Description	*P*-value	Count
KEGG_PATHWAY	hsa04723	Retrograde endocannabinoid signaling	0.004813647	8
KEGG_PATHWAY	hsa05165	Human papillomavirus infection	0.018148818	11
KEGG_PATHWAY	hsa04530	Tight junction	0.031975382	7
KEGG_PATHWAY	hsa00230	Purine metabolism	0.034383145	6
KEGG_PATHWAY	hsa04728	Dopaminergic synapse	0.038482344	6

KEGG, Kyoto Encyclopedia of Genes and Genomes.

### Identification of differentially expressed genes in Alzheimer’s disease

**FIGURE 5:**
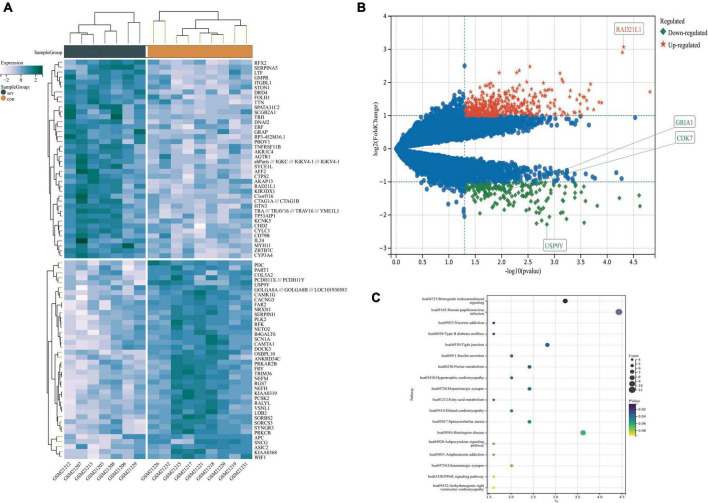
The volcano plot, heat map, and pathway’s bubble diagram of DEGs. **(A)** A heat map of DEGs in GSE1297. **(B)** Volcano plot of DEGs in GSE1297. **(C)** Pathway bubble diagram of DEGs screened by weighted correlation network analysis (WGCNA). There are also two horizontal dashed lines in the panel **(B)**, representing log2FC at –1 and 1, The vertical dashed line represents the adjusted *p*-value at 0.05, RAD21L1 and USP9Y represented the genes with the largest and smallest difference, respectively, GRIA1 and CDK7 are the key genes in this study. DEGs, differentially expressed genes.

**FIGURE 6:**
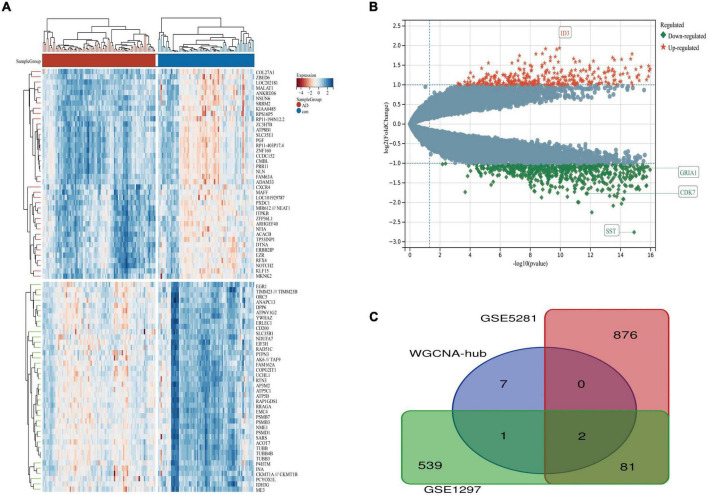
The volcano plot, heat map, and Venn diagram of DEGs. **(A)** A heat map of DEGs in GSE5281. **(B)** Volcano plot of DEGs in GSE5281. **(C)** Venn diagram of GSE5281, GSE1297, and WGCNA-hub. There are also two horizontal dashed lines in the panel **(B)**, representing log2FC at –1 and 1, The vertical dashed line represents the adjusted *p*-value at 0.05, ID3 and SST represented the genes with the largest and smallest difference, respectively. GRIA1 and CDK7 are the key genes in this study.

We downloaded the series GSE1297 and GSE5281 datasets about AD from the NCBI GEO database. After using the “limma” package in R software, screening with the threshold of an adjusted *p*-value < 0.05 and | log2FC| > 1.0, 959 DEGs (320 upregulated and 639 downregulated) were identified in the GSE5281 dataset and DEGs were identified by comparing AD samples with normal samples. 622 DEGs (408 upregulated and 215 downregulated) were identified in the GSE1297 dataset, the DEGs were identified by comparing “control” with the “Severe” status of AD samples. The volcano plot and heatmap analyses were used to visualize the DEGs of the two data sets shown in Figures 5A,B, 6A,B, respectively. In addition, a Venn diagram analysis was performed to evaluate the common DEGs between GSE5281, WGCNA-hub, and GSE1297. As presented in Figure 6C, Intersecting, with 10 hub genes identified two overlapping DEGs (CDK7 and GRIA1).

### Building protein-protein interaction networks and screening 10 key genes

**FIGURE 7:**
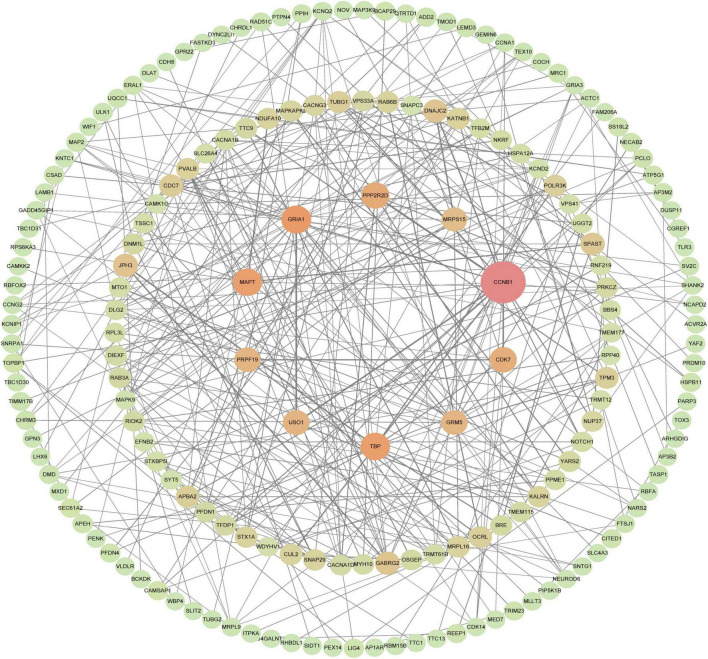
PPI network of hub genes, PPI network of genes constructed in the STRING database. Then, visualization of a STRING-derived network of molecular interactions in Cytoscape pathway visualization and analysis software, the 10 genes in the middle are the key genes to be screened, and the closer the color is to red, the higher the score.

A total of 177 nodes and 343 edges were involved in the final PPI network from 269 proteins using the STRING tool, as presented in Figure 7. The top 10 genes evaluated by the algorithm Between Centrality were adopted in the final PPI network. The results showed that the protein, a regulatory protein involved in mitosis (CCNB1), was the most outstanding one, with a score = 10,430, followed by the predominant excitatory neurotransmitter receptors in the mammalian brain which are activated in a variety of normal neurophysiologic processes (GRIA1; score = 4,243). The TFIID basal transcription factor complex plays a major role in the initiation of RNA polymerase II (Pol II)-dependent transcription. Other proteins are TATA-binding protein (TBP; score = 4,035), the microtubule-associated protein, tau (MAPT; score = 4,000), Protein Phosphatase 2 Regulatory Subunit Bdelta (PPP2R2D; score = 3,479), Cyclin-Dependent Kinase 7, involved in cell cycle control and RNA polymerase II-mediated RNA transcription (CDK7; score = 3,158), USO1 Vesicle Transport Factor (USO1; score = 2,905), a gene essential for cell survival and DNA repair (PRPF19; score = 2,809), The protein is a metabotropic glutamate receptor, whose signaling activates a phosphatidylinositol-calcium second messenger system. This protein may be involved in the regulation of neural network activity and synaptic plasticity (GRM5; score = 2,800) and Mitochondrial Ribosomal Protein S15 (MRPS15; score = 2,510). Table 3 shows the top 10 hub genes.

**TABLE 3:** The hub genes.

Rank	Name	Score
1	CCNB1	10429.51
2	GRIA1	4242.677
3	TBP	4035.28
4	MAPT	4000.436
5	PPP2R2D	3479.117
6	CDK7	3157.932
7	USO1	2904.839
8	PRPF19	2809.138
9	GRM5	2799.903
10	MRPS15	2510.792

### Prediction of microRNAs and identification of common differentially expressed miRNAs

**FIGURE 8:**
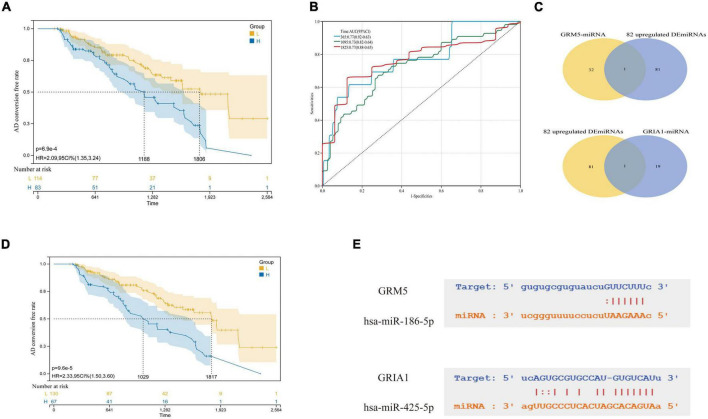
Selection of key miRNAs. **(A)** AD conversion curves of hsa-miR-186-5p. **(B)** ROC analyses for the 365, 1,095, and 1,825 time points were performed using the ROC function of the R package proc (version 1.17.0.1). **(C)** The intersection of predicted miRNAs and abnormal miRNAs in blood. **(D)** hsa-miR-425-5p. **(E)** They are, respectively, the complementary base sequence matching of hsa-miR-186-5p and target gene GRM5, and the complementary base sequence matching of hsa-miR-425-5p and target gene GRIA1.

In addition, we explored the predicted miRNAs of GRM5 and CRIA1 in AD patients using the ENCORI platform to establish potential miRNA messenger RNA (mRNA) regulatory networks (Wu et al., 2021). We found 53 predicted differentially expressed microRNAs (Figure 4C). Also, 140 specifically dysregulated differentially expressed miRNAs (DEmiRNAs) (58 downregulated and 82 upregulated DEmiRNAs) in blood samples of patients with AD were identified and used in our work, according to the report by Leidinger et al. (2013; Supplementary Data 2). The DEmiRNAs from AD were intersected with the 82 upregulated DEmiRNAs from blood samples; the common DEmiRNAs were identified. Respectively, hsa-miR-186-5p and hsa-miR-425-5p were identified to be potentially involved in the regulation of GRM5 and GRIA1 (Figures 8C,E).

### Prediction of transcription factor binding to target genes

We downloaded the TBP’s primary structure of the amino acid sequence from the Uniprot database,^8^ and then found the TBP transcription factor binding site features in the JASPAR database^9^ (Figure 4A). Then, 1,000 bp promoter regions of GRM5 and GRIA1 sequences were downloaded from the UCSC database^10^ (Supplementary Data 3). Finally, their promoter sequences were entered into the JASPAR database to predict regulatory transcription factors. Even when the threshold was set as 80%, TBP could be found to be the transcription factor of both. For a more accurate prediction of transcription factors, we used the HumanTFDB database.^11^ Interestingly, not only TBP is a transcription factor of GRM5 and GRIA1, but CDK7 is also a transcription factor of both (detailed results are provided in the Supplementary Data 4).

### Drug selection results

Fifteen drugs targeting the four core genes were selected based on drug and target information contained in DrugBank database. Of these, nine medications have been approved, six are investigational and experimental. All four GRIA1 drugs are primarily responsible for the activity of AMPA glutamate receptors and extracellular glutamate gated ion channels. Isoflurane (DB00753), Methodflurane (DB01028), Desflurane (DB01189), Sevoflurane (DB01236) are all known to be an antagonist medication. Isoflurane is a general inhaled cosmetic used in surgery. Methoxyflurane is a general inhalational anesthetic used for the induction and maintenance of general anesthesia. Desflurane is a general inhalational anesthetic for both inpatient and outpatient surgery in adults. Sevoflurane is an inhalational anesthetic agent used for the induction and maintenance of general anesthesia during surgical interventions. Additional information on the features and specific usage of other gene-targeted drugs is presented in Supplementary Data 6 and in Table 4.

**TABLE 4:** Fifteen drugs targeting key genes screened from DrugBank database.

genes	Drug name and ID	Status	Type
TBP	Quercetin (DB04216)	Experimental, investigational	
TBP	Chloroquine (DB00608)	Approved, investigational, vet_approved	Inhibitor
TBP	Revusiran (DB16309)	Investigational	Regulator
CDK7	Phosphonothreonine (DB02482)	Experimental	
CDK7	Alvocidib (DB03496)	Experimental, investigational	
CDK7	SNS-032 (DB05969)	Investigational	
CDK7	Seliciclib (DB06195)	Investigational	
CDK7	Trilaciclib (DB15442)	Approved, investigational	Inhibitor
GRIA1	Isoflurane (DB00753)	Approved, vet_approved	Antagonist
GRIA1	Methoxyflurane (DB01028)	Approved, investigational, vet_approved	Antagonist
GRIA1	Desflurane (DB01189)	Approved	Antagonist
GRIA1	Sevoflurane (DB01236)	Approved, vet_approved	Antagonist
GRM5	Imipramine (DB00458)	Approved	Inhibitor
GRM5	Disopyramide (DB00280)	Approved	Inhibitor
GRM5	Dalfampridine (DB06637)	Approved	Antagonist

## Discussion

**FIGURE 9:**
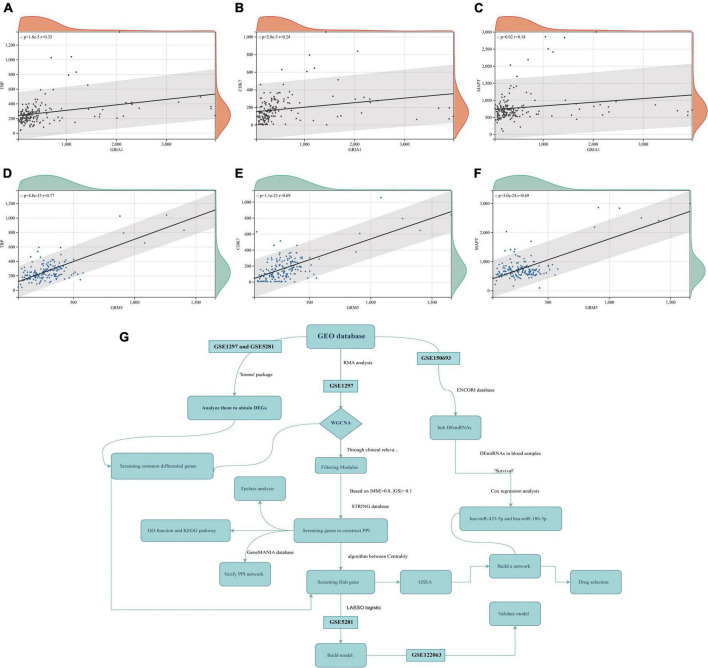
Protein-to-protein correlation graph (GSE5281 data set). **(A–C)** Respectively, the correlation map of GRIA1 and TBP, CDK7, MAPT. **(D–F)** Respectively, the correlation map of GRM5 and TBP, CDK7, MAPT. **(G)** The flow-process diagram.

Due to the heterogeneity of AD pathology, there is a lack of sufficient efficacy in treating AD (Lam et al., 2013; Byun et al., 2015). In the past decades, neurodegenerative therapy for AD has made more and more progress, and drug resistance is often allowed in traditional histology (Nandigam, 2008). Hence, identifying more appropriate molecular regulation of transcriptional clusters is critical to guiding personalized AD treatment. In this work, the top 7,200 genes based on MAD values were chosen for WGCNA analysis. The “Midnight” module of GSE1297 was chosen as it is significantly associated with clinical AD (Figure 2). Subsequently, according to the cytoHubba plug-in of Cytoscape, we made use of the algorithm Between Centrality to screen 10 key DEGs as hub genes in the PPI network, including CCNB1, GRIA1, TBP, MAPT, PPP2R2D, CDK7, USO1, PRPF19, GRM5, and MRPS15. It’s suggesting that these genes may play important role in the mechanism of AD and the specific flow chart is shown in Figure 9G.

Luckily, we discovered that in GO analysis, GRM5 and GRIA1 were predominantly enhanced in the synaptic membrane of CC (cellular component), Neuron to Neuron synapse, postsynaptic membrane, postsynaptic density, and postsynaptic specialization. In BP (biological process), GRM5 and GRIA1 were predominantly enriched in the modulation of chemical synaptic transmission, regulation of trans-synaptic signaling, and regulation of membrane potential. More and more studies have shown that neuron synapse and AD are closely related, synapse loss and Tau pathology are hallmarks of AD and other tauopathies (Yoshiyama et al., 2007; Skaper et al., 2017; Dejanovic et al., 2018; Kurucu et al., 2022). In KEGG pathway analysis, GRM5 and GRIA1 are co-enriched in glutamatergic synapse and retrograde endocannabinoid signaling pathways. TBP, GRM5, and GRIA1 were co-enriched in Huntington’s disease pathway. Both Huntington’s disease (HD) and Alzheimer’s disease (AD) are similar, albeit clinically distinct, neurodegenerative disorders that share pathologic features related to selective brain injury (Naia et al., 2017; Mukherjee, 2021). TBP and CDK7 are basal transcription factors among them. In addition, through FpClass analysis, we know that TBP and CDK7 have high reliability based on gene expression and topological network analysis, and GRM5 and GRIA1 also have high scores (Supplementary Data 7). In the verification in the GeneMANIA database, they still have the same relationship network (Supplementary Data 5, Figure A).

All three nuclear RNA polymerases have a subunit of the TATA binding protein (TBP) complex required for transcription (Davidson et al., 2004). In all fields of life, transcription regulation by DNA-dependent three-types RNA polymerase is largely achieved at the initial level. Among them, TBP is the most conservative initiation factor, which is crucial to the transcription initiation of all ancient eukaryotes, and is the only initiation factor required for the complete start of RNA polymerase in all eukaryotes (Kramm et al., 2019; Mishal and Luna-Arias, 2022). Back in 2004, they demonstrated significant differences in TBP between the AD group of normal subjects and patients. In addition, their number and distribution are not proportional to the number and the distribution of positive tau or β-amyloid structures, and the TBP accumulates in AD brains, suggesting that TBP might be a contributing factor to AD due to its own entanglements (Reid et al., 2004). Evidence implicating TBP in the molecular mechanism of several neurodegenerative diseases has emerged in the past few years. TBP may contribute to these diseases through a loss of normal function (likely to be catastrophic to a cell) which affects neurodegenerative diseases, such as AD and Huntington’s disease (van Roon-Mom et al., 2005; Bech et al., 2010).

The transcription cycle of RNA polymerase II is regulated by a group of cyclin dependent kinases (CDK). CDK7 related to TFIIH, a transcription initiation factor, is not only an effector of RNA polymerase II phosphorylation and other targets in the transcription mechanism, but also a CDK activated kinase involved in transcription, and also plays a key role in regulating eukaryotic cell division (Sansó and Fisher, 2013; Fisher, 2019). There is growing evidence that CDK regulates the transcriptional cycle of RNA polymerase II. Specific CDKs are considered as important molecular mechanisms in the transcription cycle, and describe that recently emerged transcriptional CDK can serve as promising drug targets in cancer (Fisher, 2017; Parua and Fisher, 2020). Moreover, some studies show that neuronal activity of CDK7 in hippocampus is related to aging and AD (Zhu et al., 2000). CDK7 is also required for activity-Dependent expression of Neuronal genes, synaptic plasticity at long-term, and memory at long-term (He et al., 2017). It can be hypothesized that CDK7 is a promising drug target for AD.

Although GRM5 (coding for metabotropic glutamate receptor 5, mGluR5) is a promising target for treating cognitive deficits in schizophrenia and AD, its association with cognitive and brain phenotypes within this disorder has received little attention. Early researchers found that the common genetic variation of GRM5 in schizophrenic patients would affect cognitive function, hippocampal volume and hippocampal mGluR5 protein level compared with the healthy control group, among them, the metabotropic glutamate receptor subtype 5 (mGluR5) is encoded by GRM5 gene, which is an attractive new drug target for the treatment of schizophrenia (Matosin et al., 2018). MGluR5 is a postsynaptic G-protein coupled glutamate receptor, which is closely related to several key cellular processes destroyed in schizophrenia. In preclinical models of schizophrenia, positive mGluR5 modulators have shown encouraging therapeutic potential, especially in the treatment of cognitive dysfunctions (Matosin et al., 2017). Early in the course of AD inhibition, some have attempted to do so by downregulating cholinergic receptors and glutamate receptors, which, as the disease progresses, play key roles in inflammation and oxidative stress, thereby participating in the neurodegenerative process. Accumulating evidence suggests that perturbation of the excitatory amino acid L-glutamate (L-Glu) in systems may underlie the pathogenesis of hypoxia ischemia, epilepsy, and chronic neurodegenerative diseases (e.g., Huntington’s disease and AD) (Hynd et al., 2004; Schaeffer and Gattaz, 2008). It is reported that glutamate excitatory neurotransmission is an important process in learning and memory, which is seriously damaged in AD, possibly due to β Amyloid peptides (1–42) increase in relation to oxidative stress. The researchers also found that some glutamate receptors are overactivated in AD. This sustained mild activation may lead to neuronal damage and impaired synaptic plasticity (learning). More and more evidence shows that glutamate mediated neurotoxicity is involved in the pathogenesis of AD, and this metabolic glutamate receptor (GRM5), whose signal transduction will activate the second messenger system of calcium phosphatidyl inositol, will also participate in the regulation of neural network activity and synaptic plasticity, which makes us speculate that GRM5 may be a very promising target for exploring treatment and improving AD disease (Danysz and Parsons, 2003; Doraiswamy, 2003; Tanović and Alfaro, 2006; Świetlik et al., 2022).

GluA1 (also known as GluRA or GluR1) is a glutamate receptor subunit of AMPA encoded by the GRIA1 gene, there is genome-wide association between GRIA1 and schizophrenia (Barkus et al., 2014; Ang et al., 2018). Hippocampal proteomics study has pointed out that proteins involved in neuronal excitability and synaptic plasticity (e.g., GRIA1, GRM3, and SYN1) were altered in both “normal” aging and AD (Neuner et al., 2017). Similarly, a bioinformatics study identified 464 differentially expressed genes that were modulated by silent transcription factor (REST) between AD patients and controls. REST is strongly associated with glutamatergic synapses and long-term potentiation, and among them, GRIA1 shows a significant difference in its tendency to change with REST (Xu et al., 2021). From this, we speculate GRIA1 also may be regulated by other transcription factors, thereby affecting the progression of AD disease. From RNA-Seq Expression Data from GTEx (53 Tissues, 570 Donors), the highest median expression of GRM5 and GRIA1 all in Brain—Cerebellum (Supplementary Data 5, Figure B).

A microRNA (miRNA) is a short non-coding RNA with regulatory functions in a variety of biological processes, which has been implicated in many cellular processes including cell proliferation, apoptosis, gene expression, cellular differentiation, and development (Krol et al., 2010). Many studies have shown that miRNA often binds to target mRNA through complementary sequences and induces translational inhibition or target degradation as a negative regulation of mRNA expression (Towler et al., 2015). We tried to use the ENCORI platform to predict the miRNAs that regulate GRM5 and GRIA1, respectively, and then intersected with 82 up-regulated DEmiRNAs in the blood samples of the previous experiment to determine the hub miRNAs with significant differences. Respectively, hsa-miR-186-5p and hsa-miR-425-5p were identified to be potentially involved in the regulation of GRM5 and GRIA1. Among them, As shown in Figures 8A,B,D, from the AD transformation state diagram and ROC curve, we identified two significantly up-regulated miRNAs, hsa-miR-186-5p and hsa-miR-425-5p may with good prognostic values. Recent studies have found that hsa-miR-425-5p and hsa-miR-186-5p can be used as therapeutic targets for other diseases. For example, hsa-miR-425-5p may promote tumor occurrence and metastasis by activating CTNND1 related pathway (Liu et al., 2020), hsa-miR-186-5p regulates TGFβ signaling pathway through expression suppression of SMAD7 and SMAD6 genes in colorectal cancer (Bayat et al., 2021). In our study, GRM5 and GRIA1 were found to be significantly downregulated at mRNA level and regulated by the up-regulated hsa-miR-186-5p and hsa-miR-425-5p, respectively (Figures 4D,E). It has been reported that negatively regulated miRNA-mRNA pairs contribute to the improvement of AD and provide new reliable targets for the treatment of AD (Fu et al., 2019; Qian et al., 2019; Chen et al., 2021).

**FIGURE 10:**
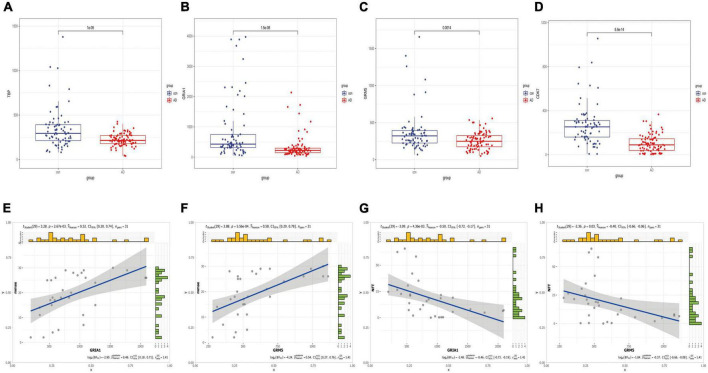
Boxplots of hub mRNA and correlation with clinical information. **(A–D)** Based on the difference boxplot of TBP, GRIA1, GRM5, and CDK7 between the normal group and AD group (GSE5281 data set). **(E–H)** The correlation between GRIA1, GRM5, and clinical information MMSE, NFT, respectively.

**FIGURE 11:**
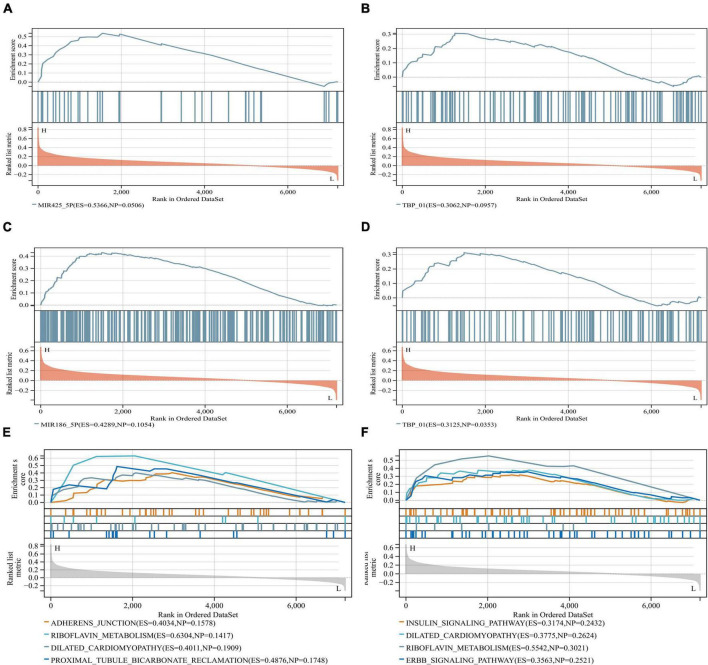
GSEA software was used to assess miRNA and TBP in similar biological pathways with target genes. **(A,B)** Grouped according to gene expression profiles and phenotypes, and evaluated related pathways and molecular mechanisms to validate the regulate of the hsa-miR-425-5p and TBP to the target gene GRIA1. **(C,D)** Validated regulate of hsa-miR-186 and TBP to the target gene GRM5, and indicate that they are on the relevant biological pathway. **(E,F)** They are GRIA1 and GRM5, respectively, and their corresponding miRNA-related biological pathways.

Anomalous aggregation of microtubule associated tau protein (MAPT) is a prominent pathological feature in various neurodegenerative diseases including AD (Zhou and Wang, 2017; Strang et al., 2019). From in Figure 9, we can observe that the correlation coefficients of GRM5 and GRIA1 with MAPT were 0.69 and 0.18, respectively, with *P* < 0.05. A reliable index of AD-related cognitive state at a particular time point is the MiniMental State Examination (MMSE) (Clark et al., 1999). Furthermore, dementia-NFT of the senile type has been termed tangle-only dementia, NFT-predominant form of SD and limbic NFT dementia (Hyman, 1997). Therefore, MMSE and NFT were chosen as our main markers to quantify AD progression (Schmitt et al., 2000; Yamada, 2003). Since NFT scores increase and MMSE scores decrease with AD severity, genes upregulated with AD could only correlate positively with NFT or negatively with MMSE, whereas genes downregulated with AD could only positively correlate with MMES or negatively correlated with NTF scores. In our study (Figure 10), the significantly down-regulated gene GRM5 was positively correlated with MMSE with a correlation coefficient of 0.58 and negatively correlated with NFT with a correlation coefficient of −0.4, GRIA1 and MMSE were also positively correlated with a correlation coefficient of 0.52 and negatively correlated with NFT with a correlation coefficient of −0.5. At the same time, their *p* < 0.05. As shown in Figures 9A–F, we also can find that the transcription factors TBP and CDK7 are well correlated with their downstream regulated genes. For the transcription factors TBP and CDK7, and the differential expression changes of their regulated target genes GRM5 and GRIA1. It can be seen from Figures 5, 6 that the expression of CDK7 and GRIA1 in AD group is two times lower than that in normal group in two databases. The expression of TBP and GRM5 also decreased significantly compared with the normal group. Grouped according to gene expression profiles and phenotypes, related pathways and molecular mechanisms were evaluated by using GSEA software to validate the regulate of hsa-miR-425-5p, hsa-miR-186, and TBP to target genes GRIA1 and GRM5, respectively (Figure 11).

**FIGURE 12:**
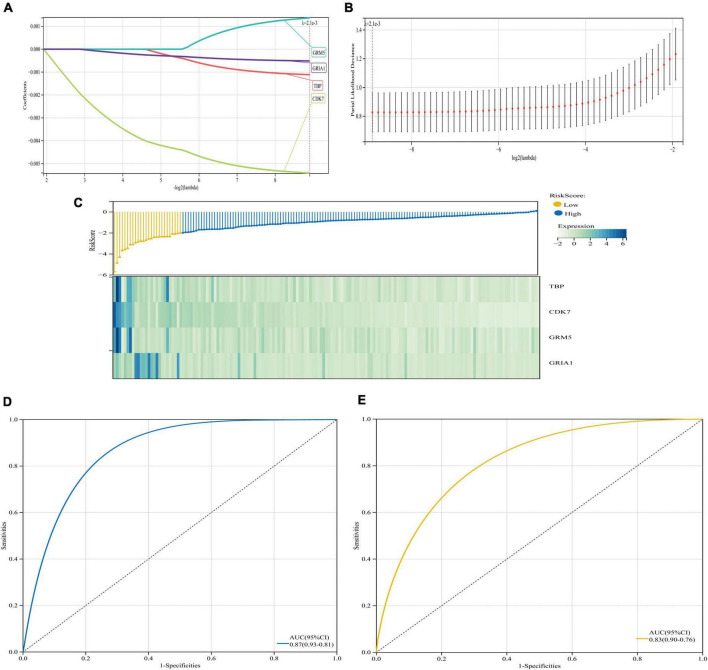
Least absolute shrinkage and selection operator (LASSO) regression algorithm and model prognostic evaluation. **(A,B)** The model based on the GSE5281 dataset with four AD-related genes was determined by Lasso-Cox algorithm. **(C)** Heatmap of risk score distribution and prognostic 4 gene signatures in the GSE5281 database. **(D,E)** ROC curve and corresponding AUC value of two databases. **(D)** Database for model building GSE5281. **(E)** Database for validation GSE122063.

We also performed regression analysis using Lasso-Cox to construct and validate a prognostic signature associated with the four key genes, which had significant predictive value for AD patients and achieved high AUC values close to 90% in both databases (Figure 12). In addition, we screened 15 drugs targeting key genes from the DrugBank database, most of which are used to relieve pain, systemic anesthetics, inhibit signaling, inhibit cyclin-dependent kinases, inhibit multiple enzyme targets (including CDK7) and change the growth phase of treated cells, treat depression, it is used in potassium channel blockers, ameliorates multiple sclerosis (MS), and so on. We can try to study these drugs, and maybe they may help us to alleviate and treat AD (Supplementary Data 7). In conclusion, based on the bioinformatics analysis in this study, we propose GRM5 and GRIA1 as novel potential prognostic markers of AD and suggest miRNAs and transcription factors that may be related to AD and regulate GRM5 and GRIA1. We hope our findings will inform future research to improve outcomes for patients and try to mitigate and treat AD.

## Conclusion

Our research identified a miRNA/TF-gene network that is potentially relevant for AD. The four hub genes (including TBP, CDK7 GRIA1, and GRM5) were markedly downregulated, which may have a critical influence on the pathophysiological mechanism of AD. Two potential target miRNAs (hsa-miR-425-5p and hsa-miR-186-5p) were furthermore forecast. The significantly down-regulated transcription factors TBP and CDK7 may be involved in the transcription of GRM5 and GRIA1, thereby affecting their expression and thus the progression of AD. TBP accumulating in the AD brain, localizing to neurofibrillary tangle structures, may be a contributing factor in AD. CDK7 is necessary for Activity-Dependent Neuronal Gene Expression, Long-Term Synaptic Plasticity, and Long-Term Memory, some studies have demonstrated that CDK7 neuronal activity in the hippocampus is linked to aging and AD. The downregulation of both TBP and CDK7 makes GRM5 and GRIA1 possible to cause neuronal damage and impaired synaptic plasticity in the pathogenesis of AD. As the metabotropic glutamate receptor encoded by GRM5 gene and the AMPA glutamate receptor subunit encoded by GRIA1 participate in the perturbed glutamatergic neurotransmission. Meanwhile, up-regulated hsa-miR-425-5p and hsa-miR-186-5p inhibited the transcription of GRIA1 and GRM5 by binding to them. These findings may contribute to the early diagnostic strategies, treatment targets and prognostic markers of AD disease.

## Data availability statement

The original contributions presented in this study are included in the article/Supplementary material, further inquiries can be directed to the corresponding authors.

## Author contributions

QZ and PY designed the research study and wrote the original article. QZ, WG, and PY performed the data analysis. QZ, XP, YS, YW, and CP participated in the conception of the study and revision of the manuscript. YW was responsible for guiding the research framework, including concepts and theories. All authors approved the final version to be published.

## Footnotes

1
https://www.ncbi.nlm.nih.gov/geo/browse/

2
https://david.ncifcrf.gov/

3
www.cytoscape.org/

4
http://software.broadinstitute.org/gsea/index.jsp

5
http://www.gsea-msigdb.org/gsea/downloads.jsp

6
www.DrugBank.ca

7
https://www.ncbi.nlm.nih.gov/geo/query/acc.cgi?acc=GSE1297

8
https://www.uniprot.org/

9
https://jaspar.genereg.net/

10
http://genome.ucsc.edu/

11
http://bioinfo.life.hust.edu.cn/HumanTFDB#!/

## References

[B1] Andreasen N. Minthon L. Vanmechelen E. Vanderstichele H. Davidsson P. Winblad B. (1999). Cerebrospinal fluid tau and Aβ42 as predictors of development of Alzheimer’s disease in patients with mild cognitive impairment. *Neurosci. Lett.* 273 5–8. 10.1016/S0304-3940(99)00617-5 10505638

[B2] Ang G. McKillop L. E. Purple R. Blanco-Duque C. Peirson S. N. Foster R. G. (2018). Absent sleep EEG spindle activity in GluA1 (Gria1) knockout mice: Relevance to neuropsychiatric disorders. *Transl. Psychiatry* 8:154. 10.1038/s41398-018-0199-2 30108203 PMC6092338

[B3] Arvanitakis Z. Bennett D. A. (2019). What is dementia?. *Jama* 322 1728–1728. 10.1001/jama.2019.11653 31688886 PMC7455009

[B4] Arvanitakis Z. Shah R. C. Bennett D. A. (2019). Diagnosis and management of dementia. *Jama* 322 1589–1599. 10.1001/jama.2019.4782 31638686 PMC7462122

[B5] Bakota L. Brandt R. (2016). Tau Biology and Tau-Directed Therapies for Alzheimer’s Disease. *Drugs* 76 301–313. 10.1007/s40265-015-0529-0 26729186 PMC4757605

[B6] Barkus C. Sanderson D. J. Rawlins J. N. Walton M. E. Harrison P. J. Bannerman D. M. (2014). What causes aberrant salience in schizophrenia? A role for impaired short-term habituation and the GRIA1 (GluA1) AMPA receptor subunit. *Mol. Psychiatry* 19 1060–1070. 10.1038/mp.2014.91 25224260 PMC4189912

[B7] Bayat Z. Ghaemi Z. Behmanesh M. Soltani B. M. (2021). Hsa-miR-186-5p regulates TGFβ signaling pathway through expression suppression of SMAD6 and SMAD7 genes in colorectal cancer. *Biol. Chem.* 402 469–480. 10.1515/hsz-2019-0407 33938174

[B8] Bech S. Petersen T. Nørremølle A. Gjedde A. Ehlers L. Eiberg H. (2010). Huntington’s disease-like and ataxia syndromes: Identification of a family with a de novo SCA17/TBP mutation. *Parkinsonism Relat. Disord.* 16 12–15. 10.1016/j.parkreldis.2009.06.006 19595623

[B9] Blalock E. M. Geddes J. W. Chen K. C. Porter N. M. Markesbery W. R. Landfield P. W. (2004). Incipient Alzheimer’s disease: Microarray correlation analyses reveal major transcriptional and tumor suppressor responses. *Proc. Natl. Acad. Sci. U. S. A.* 101 2173–2178. 10.1073/pnas.0308512100 14769913 PMC357071

[B10] Byun M. S. Kim S. E. Park J. Yi D. Choe Y. M. Sohn B. K. (2015). Heterogeneity of Regional Brain Atrophy Patterns Associated with Distinct Progression Rates in Alzheimer’s Disease. *PLoS One* 10:e0142756. 10.1371/journal.pone.0142756 26618360 PMC4664412

[B11] Chen M. L. Hong C. G. Yue T. Li H. M. Duan R. Hu W. B. (2021). Inhibition of miR-331-3p and miR-9-5p ameliorates Alzheimer’s disease by enhancing autophagy. *Theranostics* 11 2395–2409. 10.7150/thno.47408 33500732 PMC7797673

[B12] Chong F. P. Ng K. Y. Koh R. Y. Chye S. M. (2018). Tau Proteins and Tauopathies in Alzheimer’s Disease. *Cell. Mol. Neurobiol.* 38 965–980. 10.1007/s10571-017-0574-1 29299792 PMC11481908

[B13] Clark C. M. Sheppard L. Fillenbaum G. G. Galasko D. Morris J. C. Koss E. (1999). Variability in annual Mini-Mental State Examination score in patients with probable Alzheimer disease: A clinical perspective of data from the Consortium to Establish a Registry for Alzheimer’s Disease. *Arch. Neurol.* 56 857–862. 10.1001/archneur.56.7.857 10404988

[B14] Corbo C. P. Alonso Adel C. (2011). Therapeutic targets in Alzheimer’s disease and related tauopathies. *Prog. Mol. Biol. Transl. Sci.* 98 47–83. 10.1016/b978-0-12-385506-0.00002-8 21199770

[B15] Danysz W. Parsons C. G. (2003). The NMDA receptor antagonist memantine as a symptomatological and neuroprotective treatment for Alzheimer’s disease: Preclinical evidence. *Int. J. Geriatr. Psychiatry* 18 S23–S32. 10.1002/gps.938 12973747

[B16] Davidson I. Martianov I. Viville S. (2004). TBP, a universal transcription factor?. *Med. Sci.* 20 575–579. 10.1051/medsci/2004205575 15190478

[B17] Dejanovic B. Huntley M. A. De Mazière A. Meilandt W. J. Wu T. Srinivasan K. (2018). Changes in the Synaptic Proteome in Tauopathy and Rescue of Tau-Induced Synapse Loss by C1q Antibodies. *Neuron* 100 1322–1336.e7. 10.1016/j.neuron.2018.10.014 30392797

[B18] Doraiswamy P. M. (2003). Alzheimer’s disease and the glutamate NMDA receptor. *Psychopharmacol. Bull.* 37 41–49. 14566213

[B19] Du Y. Liu X. Zhu X. Liu Y. Wang X. Wu X. (2020). Activating transcription factor 6 reduces Aβ1-42 and restores memory in Alzheimer’s disease model mice. *Int. J. Neurosci.* 130 1015–1023. 10.1080/00207454.2020.1715977 31928492

[B20] Esteller M. (2011). Non-coding RNAs in human disease. *Nat. Rev. Genet.* 12 861–874. 10.1038/nrg3074 22094949

[B21] Fisher R. P. (2017). CDK regulation of transcription by RNAP II: Not over ‘til it’s over? *Transcription* 8 81–90. 10.1080/21541264.2016.1268244 28005463 PMC5423476

[B22] Fisher R. P. (2019). Cdk7: A kinase at the core of transcription and in the crosshairs of cancer drug discovery. *Transcription* 10 47–56. 10.1080/21541264.2018.1553483 30488763 PMC6602562

[B23] Fu Y. Hu X. Zheng C. Sun G. Xu J. Luo S. (2019). Intrahippocampal miR-342-3p inhibition reduces β-amyloid plaques and ameliorates learning and memory in Alzheimer’s disease. *Metab. Brain Dis.* 34 1355–1363. 10.1007/s11011-019-00438-9 31134481

[B24] Geng Y. N. Wu Y. J. Zhang W. X. (2016). Effect of hyperforin on learning and memory abilities and Aβ_*1–42*_, βAPP and BACE1 protein expressions in hippocampus of Alzheimer’s disease model mice. *Zhongguo Zhong Yao Za Zhi* 41 2877–2882. 10.4268/cjcmm20161522 28914032

[B25] Hara N. Kikuchi M. Miyashita A. Hatsuta H. Saito Y. Kasuga K. (2017). Serum microRNA miR-501-3p as a potential biomarker related to the progression of Alzheimer’s disease. *Acta Neuropathol. Commun.* 5:10. 10.1186/s40478-017-0414-z 28137310 PMC5282710

[B26] He G. Yang X. Wang G. Qi J. Mao R. Wu Z. (2017). Cdk7 Is Required for Activity-Dependent Neuronal Gene Expression, Long-Lasting Synaptic Plasticity and Long-Term Memory. *Front. Mol. Neurosci.* 10:365. 10.3389/fnmol.2017.00365 29163040 PMC5681959

[B27] Hyman B. T. (1997). The neuropathological diagnosis of Alzheimer’s disease: Clinical-pathological studies. *Neurobiol. Aging* 18 S27–S32. 10.1016/s0197-4580(97)00066-3 9330982

[B28] Hynd M. R. Scott H. L. Dodd P. R. (2004). Glutamate-mediated excitotoxicity and neurodegeneration in Alzheimer’s disease. *Neurochem. Int.* 45 583–595. 10.1016/j.neuint.2004.03.007 15234100

[B29] Jäkel L. De Kort A. M. Klijn C. J. Schreuder F. H. Verbeek M. M. (2022). Prevalence of cerebral amyloid angiopathy: A systematic review and meta-analysis. *Alzheimers Dement.* 18 10–28. 10.1002/ALZ.12366 34057813 PMC9290643

[B30] Kotlyar M. Pastrello C. Pivetta F. Lo Sardo A. Cumbaa C. Li H. (2015). In silico prediction of physical protein interactions and characterization of interactome orphans. *Nat. Methods* 12 79–84. 10.1038/nmeth.3178 25402006

[B31] Kramm K. Engel C. Grohmann D. (2019). Transcription initiation factor TBP: Old friend new questions. *Biochem. Soc. Trans.* 47 411–423. 10.1042/bst20180623 30710057

[B32] Krol J. Loedige I. Filipowicz W. (2010). The widespread regulation of microRNA biogenesis, function and decay. *Nat. Rev. Genet.* 11 597–610. 10.1038/nrg2843 20661255

[B33] Kumar S. Reddy P. H. (2019). A New Discovery of MicroRNA-455-3p in Alzheimer’s Disease. *J. Alzheimers Dis.* 72 S117–S130. 10.3233/jad-190583 31524168

[B34] Kumar S. Vijayan M. Reddy P. H. (2017). MicroRNA-455-3p as a potential peripheral biomarker for Alzheimer’s disease. *Hum. Mol. Genet.* 26 3808–3822. 10.1093/hmg/ddx267 28934394 PMC6075184

[B35] Kurucu H. Colom-Cadena M. Davies C. Wilkins L. King D. Rose J. (2022). Inhibitory synapse loss and accumulation of amyloid beta in inhibitory presynaptic terminals in Alzheimer’s disease. *Eur. J. Neurol.* 29 1311–1323. 10.1111/ene.15043 34331352

[B36] Lam B. Masellis M. Freedman M. Stuss D. T. Black S. E. (2013). Clinical, imaging, and pathological heterogeneity of the Alzheimer’s disease syndrome. *Alzheimers Res. Ther.* 5:1. 10.1186/alzrt155 23302773 PMC3580331

[B37] Leidinger P. Backes C. Deutscher S. Schmitt K. Mueller S. C. Frese K. (2013). A blood based 12-miRNA signature of Alzheimer disease patients. *Genome Biol.* 14:R78. 10.1186/gb-2013-14-7-r78 23895045 PMC4053778

[B38] Liang W. S. Dunckley T. Beach T. G. Grover A. Mastroeni D. Walker D. G. (2007). Gene expression profiles in anatomically and functionally distinct regions of the normal aged human brain. *Physiol. Genom.* 28 311–322. 10.1152/physiolgenomics.00208.2006 17077275 PMC2259385

[B39] Lin Y. Liang X. Yao Y. Xiao H. Shi Y. Jingxian (2022). Retraction notice to “Osthole attenuates APP-induced Alzheimer’s disease through up-regulating miRNA-101a-3p” [Life Sci. 225 (2019) 117-131]. *Life Sci.* 305:120747. 10.1016/j.lfs.2022.120747 35840415

[B40] Liu D. Zhang H. Cui M. Chen C. Feng Y. (2020). Hsa-miR-425-5p promotes tumor growth and metastasis by activating the CTNND1-mediated β-catenin pathway and EMT in colorectal cancer. *Cell Cycle* 19 1917–1927. 10.1080/15384101.2020.1783058 32594834 PMC7469528

[B41] Long J. M. Holtzman D. M. (2019). Alzheimer disease: An update on pathobiology and treatment strategies. *Cell* 179 312–339. 10.1016/j.cell.2019.09.001 31564456 PMC6778042

[B42] Matosin N. Fernandez-Enright F. Lum J. S. Newell K. A. (2017). Shifting towards a model of mGluR5 dysregulation in schizophrenia: Consequences for future schizophrenia treatment. *Neuropharmacology* 115 73–91. 10.1016/j.neuropharm.2015.08.003 26349010

[B43] Matosin N. Newell K. A. Quidé Y. Andrews J. L. Teroganova N. Green M. J. (2018). Effects of common GRM5 genetic variants on cognition, hippocampal volume and mGluR5 protein levels in schizophrenia. *Brain Imaging Behav.* 12 509–517. 10.1007/s11682-017-9712-0 28405888

[B44] Mayeux R. P. Stern Y. (2012). Epidemiology of Alzheimer Disease. *Cold Spring Harb. Prespect. Med.* 8:a006239. 10.1101/cshperspect.a006239 22908189 PMC3405821

[B45] Mishal R. Luna-Arias J. P. (2022). Role of the TATA-box binding protein (TBP) and associated family members in transcription regulation. *Gene* 833:146581. 10.1016/j.gene.2022.146581 35597524

[B46] Moloney C. M. Lowe V. J. Murray M. E. (2021). Visualization of neurofibrillary tangle maturity in Alzheimer’s disease: A clinicopathologic perspective for biomarker research. *Alzheimers Dement.* 17 1554–1574. 10.1002/ALZ.12321 33797838 PMC8478697

[B47] Mukherjee S. (2021). Immune gene network of neurological diseases: Multiple sclerosis (MS), Alzheimer’s disease (AD), Parkinson’s disease (PD) and Huntington’s disease (HD). *Heliyon* 7:e08518. 10.1016/j.heliyon.2021.e08518 34926857 PMC8649734

[B48] Naia L. Ferreira I. L. Ferreiro E. Rego A. C. (2017). Mitochondrial Ca(2+) handling in Huntington’s and Alzheimer’s diseases - Role of ER-mitochondria crosstalk. *Biochem. Biophys. Res. Commun.* 483 1069–1077. 10.1016/j.bbrc.2016.07.122 27485547

[B49] Nandigam R. N. (2008). Mixed brain pathologies account for most dementia cases in community-dwelling older persons. *Neurology* 70:816. 10.1212/01.wnl.0000307675.38908.39 18316696

[B50] Neuner S. M. Wilmott L. A. Hoffmann B. R. Mozhui K. Kaczorowski C. C. (2017). Hippocampal proteomics defines pathways associated with memory decline and resilience in normal aging and Alzheimer’s disease mouse models. *Behav. Brain Res.* 322 288–298. 10.1016/j.bbr.2016.06.002 27265785 PMC5135662

[B51] Osama A. Zhang J. Yao J. Yao X. Fang J. (2020). Nrf2: A dark horse in Alzheimer’s disease treatment. *Ageing Res. Rev.* 64:101206. 10.1016/j.arr.2020.101206 33144124

[B52] Parua P. K. Fisher R. P. (2020). Dissecting the Pol II transcription cycle and derailing cancer with CDK inhibitors. *Nat. Chem. Biol.* 16 716–724. 10.1038/s41589-020-0563-4 32572259 PMC7914107

[B53] Qian Q. Zhang J. He F. P. Bao W. X. Zheng T. T. Zhou D. M. (2019). Down-regulated expression of microRNA-338-5p contributes to neuropathology in Alzheimer’s disease. *Faseb J.* 33 4404–4417. 10.1096/fj.201801846R 30576233 PMC6404576

[B54] Reid S. J. van Roon-Mom W. M. Wood P. C. Rees M. I. Owen M. J. Faull R. L. (2004). TBP, a polyglutamine tract containing protein, accumulates in Alzheimer’s disease. *Brain Res. Mol. Brain Res.* 125 120–128. 10.1016/j.molbrainres.2004.03.018 15193429

[B55] Sand M. Gambichler T. Sand D. Skrygan M. Altmeyer P. Bechara F. G. (2009). MicroRNAs and the skin: Tiny players in the body’s largest organ. *J. Dermatol. Sci.* 53 169–175. 10.1016/j.jdermsci.2008.10.004 19058951

[B56] Sansó M. Fisher R. P. (2013). Pause, play, repeat: CDKs push RNAP II’s buttons. *Transcription* 4 146–152. 10.4161/trns.25146 23756342 PMC3977912

[B57] Schaeffer E. L. Gattaz W. F. (2008). Cholinergic and glutamatergic alterations beginning at the early stages of Alzheimer disease: Participation of the phospholipase A2 enzyme. *Psychopharmacology* 198 1–27. 10.1007/s00213-008-1092-0 18392810

[B58] Schmitt F. A. Davis D. G. Wekstein D. R. Smith C. D. Ashford J. W. Markesbery W. R. (2000). Preclinical” AD revisited: Neuropathology of cognitively normal older adults. *Neurology* 55 370–376. 10.1212/wnl.55.3.370 10932270

[B59] Shao C. Y. Mirra S. S. Sait H. B. R. Sacktor T. C. Sigurdsson E. M. (2011). Postsynaptic degeneration as revealed by PSD-95 reduction occurs after advanced Aβ and tau pathology in transgenic mouse models of Alzheimer’s disease. *Acta Neuropathol.* 122 285–292. 10.1007/s00401-011-0843-x 21630115 PMC3437675

[B60] Shen W. Song Z. Zhong X. Huang M. Shen D. Gao P. (2022). Sangerbox: A comprehensive, interaction-friendly clinical bioinformatics analysis platform. *Imeta* 1:e36. 10.1002/imt2.36 PMC10989974 38868713

[B61] Shigemizu D. Akiyama S. Higaki S. Sugimoto T. Sakurai T. Boroevich K. A. (2020). Prognosis prediction model for conversion from mild cognitive impairment to Alzheimer’s disease created by integrative analysis of multi-omics data. *Alzheimers Res. Ther.* 12:145. 10.1186/s13195-020-00716-0 33172501 PMC7656734

[B62] Skaper S. D. Facci L. Zusso M. Giusti P. (2017). Synaptic Plasticity, Dementia and Alzheimer Disease. *CNS Neurol. Disord. Drug Targets* 16 220–233. 10.2174/1871527316666170113120853 28088900

[B63] Song J. X. Malampati S. Zeng Y. Durairajan S. S. K. Yang C. B. Tong B. C. (2020). A small molecule transcription factor EB activator ameliorates beta-amyloid precursor protein and Tau pathology in Alzheimer’s disease models. *Aging Cell.* 19:e13069. 10.1111/acel.13069 31858697 PMC6996953

[B64] Sriroopreddy R. Sajeed R. Raghuraman P. Sudandiradoss C. (2019). Differentially expressed gene (DEG) based protein-protein interaction (PPI) network identifies a spectrum of gene interactome, transcriptome and correlated miRNA in nondisjunction Down syndrome. *Int. J. Biol. Macromol.* 122 1080–1089. 10.1016/j.ijbiomac.2018.09.056 30218739

[B65] Strang K. H. Golde T. E. Giasson B. I. (2019). MAPT mutations, tauopathy, and mechanisms of neurodegeneration. *Lab. Invest.* 99 912–928. 10.1038/s41374-019-0197-x 30742061 PMC7289372

[B66] Swarbrick S. Wragg N. Ghosh S. Stolzing A. (2019). Systematic Review of miRNA as Biomarkers in Alzheimer’s Disease. *Mol. Neurobiol.* 56 6156–6167. 10.1007/s12035-019-1500-y 30734227 PMC6682547

[B67] Świetlik D. Białowąs J. Kusiak A. Krasny M. (2022). Virtual Therapy with the NMDA Antagonist Memantine in Hippocampal Models of Moderate to Severe Alzheimer’s Disease, in Silico Trials. *Pharmaceuticals* 15:546. 10.3390/ph15050546 35631372 PMC9145937

[B68] Szklarczyk D. Franceschini A. Wyder S. Forslund K. Heller D. Huerta-Cepas J. (2015). STRING v10: Protein-protein interaction networks, integrated over the tree of life. *Nucleic Acids Res.* 43 D447–D452. 10.1093/nar/gku1003 25352553 PMC4383874

[B69] Tanović A. Alfaro V. (2006). Glutamate-related excitotoxicity neuroprotection with memantine, an uncompetitive antagonist of NMDA-glutamate receptor, in Alzheimer’s disease and vascular dementia. *Rev. Neurol.* 42 607–616. 16703529

[B70] Thanvi B. Robinson T. (2006). Sporadic cerebral amyloid angiopathy—an important cause of cerebral haemorrhage in older people. *Age Ageing* 35 565–571. 10.1093/ageing/afl108 16982664

[B71] Toden S. Zumwalt T. J. Goel A. (2021). Non-coding RNAs and potential therapeutic targeting in cancer. *Biochim. Biophys. Acta Rev. Cancer* 1875:188491. 10.1016/j.bbcan.2020.188491 33316377 PMC7856203

[B72] Towler B. P. Jones C. I. Newbury S. F. (2015). Mechanisms of regulation of mature miRNAs. *Biochem. Soc. Trans.* 43 1208–1214. 10.1042/bst20150157 26614662

[B73] van Roon-Mom W. M. Reid S. J. Faull R. L. Snell R. G. (2005). TATA-binding protein in neurodegenerative disease. *Neuroscience* 133 863–872. 10.1016/j.neuroscience.2005.03.024 15916858

[B74] Wang X. Michaelis M. L. Michaelis E. K. (2010). Functional Genomics of Brain Aging and Alzheimer’s Disease: Focus on Selective Neuronal Vulnerability. *Curr. Genom.* 11 618–633. 10.2174/138920210793360943 21629439 PMC3078686

[B75] Wishart D. S. Feunang Y. D. Guo A. C. Lo E. J. Marcu A. Grant J. R. (2018). DrugBank 5.0: A major update to the DrugBank database for 2018. *Nucleic Acids Res.* 46 D1074–D1082. 10.1093/nar/gkx1037 29126136 PMC5753335

[B76] Wu D. Huo C. Jiang S. Huang Y. Fang X. Liu J. (2021). Exostosin1 as a novel prognostic and predictive biomarker for squamous cell lung carcinoma: A study based on bioinformatics analysis. *Cancer Med.* 10 2787–2801. 10.1002/cam4.3643 33314711 PMC8026939

[B77] Xiao Q. Yan P. Ma X. Liu H. Perez R. Zhu A. (2015). Neuronal-Targeted TFEB Accelerates Lysosomal Degradation of APP, Reducing Aβ Generation and Amyloid Plaque Pathogenesis. *J. Neurosci.* 35 12137–12151. 10.1523/jneurosci.0705-15.2015 26338325 PMC4556784

[B78] Xu C. Zhang M. Zu L. Zhang P. Sun L. Liu X. (2021). Repressor element-1 silencing transcription factor regulates glutamate receptors and immediate early genes to affect synaptic plasticity. *Aging* 13 15569–15579. 10.18632/aging.203118 34106879 PMC8221361

[B79] Xue B. Qu Y. Zhang X. Xu X. F. (2022). miRNA-126a-3p Participates in Hippocampal Memory via Alzheimer’s Disease-Related Proteins. *Cereb. Cortex* 32 4763–4781. 10.1093/cercor/bhab515 35059720

[B80] Yamada M. (2003). Senile dementia of the neurofibrillary tangle type (tangle-only dementia): Neuropathological criteria and clinical guidelines for diagnosis. *Neuropathology* 23 311–317. 10.1046/j.1440-1789.2003.00522.x 14719548

[B81] Yang C. Su C. Iyaswamy A. Krishnamoorthi S. K. Zhu Z. Yang S. (2022). Celastrol enhances transcription factor EB (TFEB)-mediated autophagy and mitigates Tau pathology: Implications for Alzheimer’s disease therapy. *Acta Pharm. Sin. B* 12 1707–1722. 10.1016/j.apsb.2022.01.017 35847498 PMC9279716

[B82] Yoshiyama Y. Higuchi M. Zhang B. Huang S. M. Iwata N. Saido T. C. (2007). Synapse loss and microglial activation precede tangles in a P301S tauopathy mouse model. *Neuron* 53 337–351. 10.1016/j.neuron.2007.01.010 17270732

[B83] Zheng X. Lin W. Jiang Y. Lu K. Wei W. Huo Q. (2021). Electroacupuncture ameliorates beta-amyloid pathology and cognitive impairment in Alzheimer disease via a novel mechanism involving activation of TFEB (transcription factor EB). *Autophagy* 17 3833–3847. 10.1080/15548627.2021.1886720 33622188 PMC8632298

[B84] Zhou F. Wang D. (2017). The associations between the MAPT polymorphisms and Alzheimer’s disease risk: A meta-analysis. *Oncotarget* 8 43506–43520. 10.18632/oncotarget.16490 28415654 PMC5522165

[B85] Zhu X. Rottkamp C. A. Raina A. K. Brewer G. J. Ghanbari H. A. Boux H. (2000). Neuronal CDK7 in hippocampus is related to aging and Alzheimer disease. *Neurobiol. Aging* 21 807–813. 10.1016/s0197-4580(00)00217-7 11124424

