# Supplementary material for: Preliminary exploration of the co-regulation of Alzheimer’s disease pathogenic genes by microRNAs and transcription factors
Source: Front Aging Neurosci. 2022 Dec 6;14:1069606. doi: 10.3389/fnagi.2022.1069606 (PMC9764863; doi:10.3389/fnagi.2022.1069606)
Supplement: Supplementary file 1 [file Data_Sheet_1.zip › Supplementary Data 1.docx]

FIGURE 1|

GO analysis results of DEGs. Panel (BP), GO biological process enrichment results; panel (CC), GO cell component enrichment results. Panel (MF), GO molecular function enrichment results. Panel (A), Each rectangle represents the fitted p-value: the redder the color, the higher the enrichment. Panel (B) and (C), The genes contained in the more enriched category, and the relationship between the classes.
