# Supplementary material for: Preliminary exploration of the co-regulation of Alzheimer’s disease pathogenic genes by microRNAs and transcription factors
Source: Front Aging Neurosci. 2022 Dec 6;14:1069606. doi: 10.3389/fnagi.2022.1069606 (PMC9764863; doi:10.3389/fnagi.2022.1069606)
Supplement: Supplementary file 1 [file Data_Sheet_1.zip › Supplementary Data 5.docx]

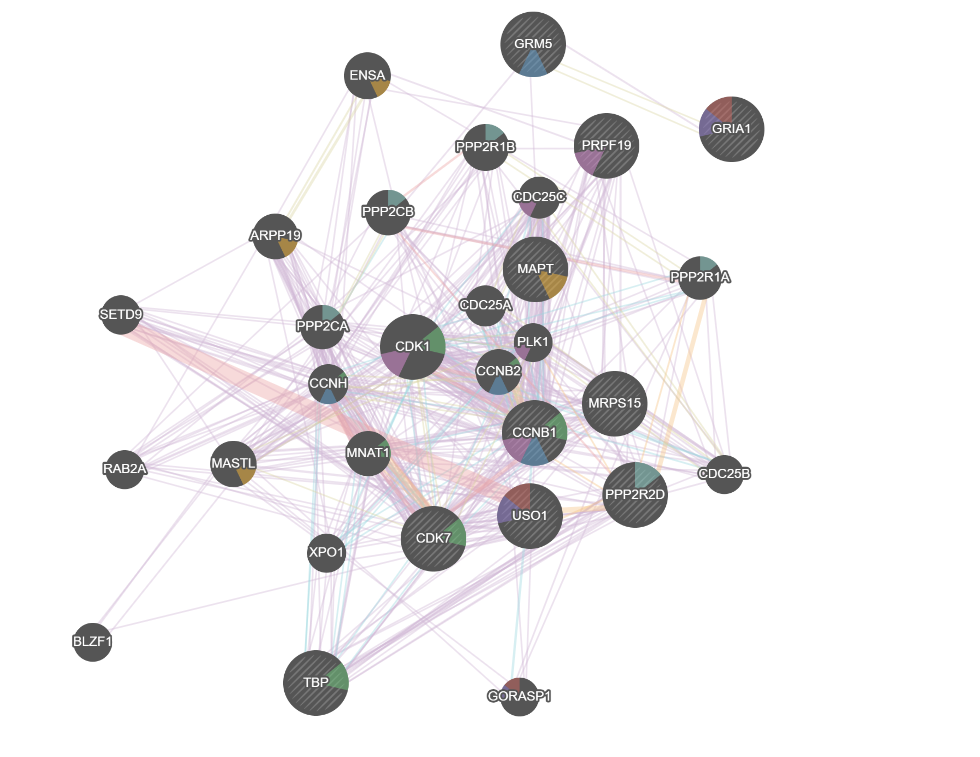


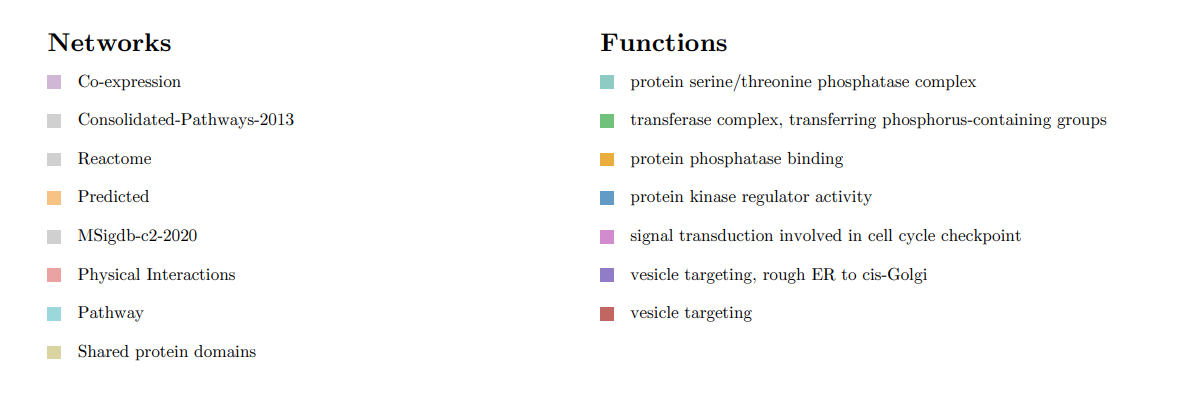


**Figure A,** Analysis of Gene Interaction Network of Hub DEGs Using GeneMANIA Database.


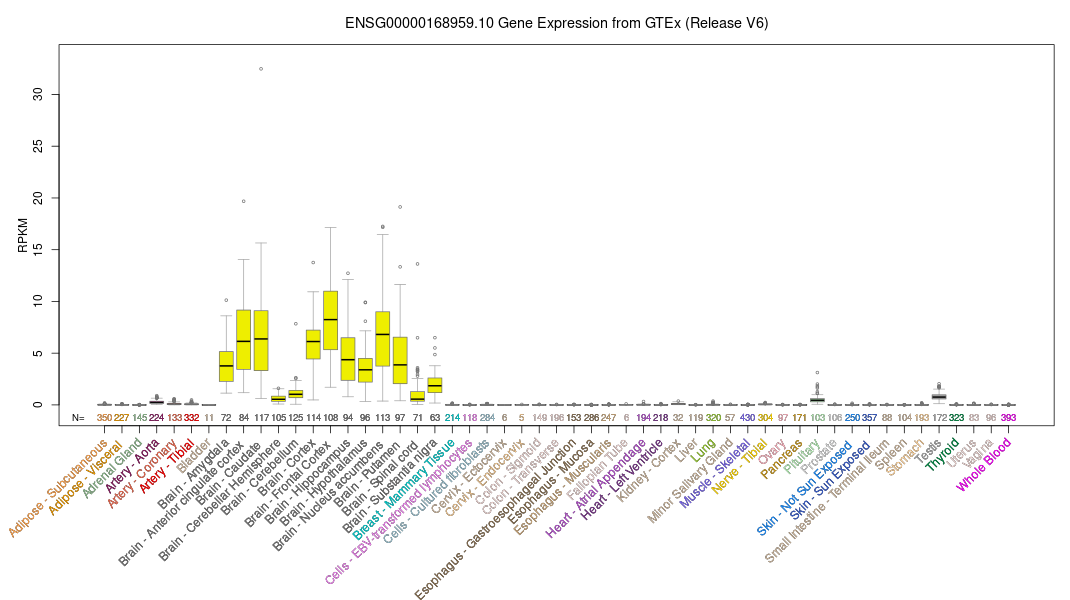


The RNA-Seq Expression Data of GRM5.


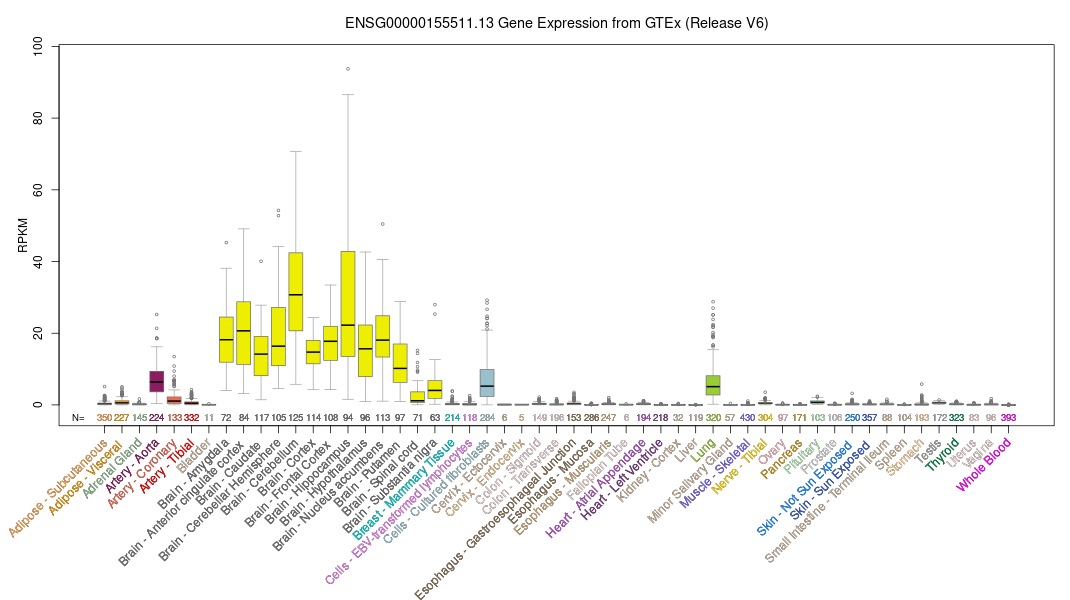


The RNA-Seq Expression Data of GRIA1.

**Figure B**, The RNA-Seq Expression Data of GRM5 and GRIA1.
